# Effects of Dietary Inclusion of Avocado Seeds on Performance, Nutrient Digestibility, Plasma Biochemical Profile, and Carcass and Meat Traits of Growing Pigs

**DOI:** 10.3390/ani15060780

**Published:** 2025-03-10

**Authors:** Consolación García-Contreras, Ana Haro, Manuel Lachica, Isabel Seiquer, Luis Lara, Ignacio Fernández-Fígares, Rosa Nieto

**Affiliations:** Department of Nutrition and Sustainable Animal Production, Estación Experimental del Zaidín, Spanish National Research Council, CSIC, Profesor Albareda, s/n, 18008 Granada, Spain; consolacion.garcia@eez.csic.es (C.G.-C.); ana.haro@eez.csic.es (A.H.); manuel.lachica@eez.csic.es (M.L.); isabel.seiquer@eez.csic.es (I.S.); luis.lara@eez.csic.es (L.L.); ignacio.fernandez-figares@eez.csic.es (I.F.-F.)

**Keywords:** avocado seed, growth, nutrient digestibility, nitrogen retention, carcass traits, energy value, pigs

## Abstract

Avocado seeds (which are discarded during fruit processing) generate residue that could be utilized in pig feeding. The objective of this study was to test the effects of dietary inclusion of dried-milled avocado seeds (DAS) on pig performance, nutrient and energy digestibility, plasma biochemical parameters, and carcass and meat traits. Twenty-four Landrace × Large White barrows (24 kg body weight, BW) were randomly allocated to three experimental treatments: control diet (CO; 18% CP, 1.12% Lys, and 14 MJ ME/kg), and two diets in which 100 or 200 g DAS/kg partially replaced a CO diet (S10 and S20, respectively). Pigs were individually housed (22 ± 1 °C), and feed and water were provided ad libitum. Animals were weighed weekly and individual intake was monitored daily. The total tract apparent digestibility (TTAD) and nitrogen balance were determined. The experiment ended at 40 kg BW, when the animals were slaughtered for blood and tissue sampling. Voluntary feed intake was not affected by the addition of up to 200 g DAS/kg to the diet. However, growth, nutrient TTAD, and nitrogen retention were depressed at the highest DAS inclusion level. The nutritional characteristics of longissimus lumborum muscle were not affected by DAS ingestion. The inclusion of up to 100 g DAS/kg in the diets of growing pigs could be used to add value to this waste product.

## Simple Summary

The sustainability of pig feeding chains can be enhanced by the inclusion of by-products from agroindustry, giving additional value to materials that are considered as waste. Avocado production has increased worldwide, and seeds are often discarded during fruit processing. We tested the inclusion of dried-milled avocado seeds in the diets of growing pigs (0, 100 and 200 g/kg). The inclusion of dried-milled seeds did not affect the voluntary feed intake of pigs. An energy value for the inclusion of dried avocado seeds in pig diets is provided (11.5 and 10.9 kJ/g dry matter of digestible and metabolizable energy, respectively). Up to 100 g/kg dried avocado seeds can be added to the practical diets of growing pigs with no deleterious effects on performance and nutrient digestibility, contributing to the value of this waste product.

## 1. Introduction

Growing public concern for the environment and animal welfare is driving the adoption of sustainable practices in animal production and the food industry. Horticultural and fruit waste—abundant in Spain and in other European countries—contain beneficial compounds that can be reused in livestock diets, promoting the circular economy [1]. By-products are non-primary products obtained from a specific process that can be used as raw materials for other processes, adding value to production chains. Traditionally, the industry has used food by-products as raw materials for animal feeds and as substrates for composting [2,3]. Avocado (*Persea americana* Mill.) is a tropical and subtropical fruit native to southern Mexico and other Mesoamerican regions [4], of which production has increased worldwide [5]. The commercial processing of the fruit results in approximately 30% of the total fruit weight being discarded as waste [6]. The avocado processing industry yields essential oils, and after processing, the pulp, seeds, peels, and exhausted pulp are discarded as waste, generating a large amount of solid residue, representing a problem for processing industries due to the significant ecological impact of their disposal in landfills [7]. In Spain, more than 115.000 t are produced annually [8], generating a considerable amount of waste. On the other hand, avocado by-products can be considered as an energy source for animals [9,10]. Avocado seeds inclusion in animal feeds has been explored, due to their potential as a source of nutrients and beneficial compounds [11]. Avocado seeds contain high fiber content, which can improve gastrointestinal health; moreover, they contain phenolic compounds and flavonoids [6], which are natural antioxidants with the potential to enhance immune health and reduce oxidative stress in animals [12]. In addition, Chia and Dykes (2020) [13] demonstrated the antimicrobial activity of ethanol extracts of avocado seeds against selected Gram-positive and Gram-negative bacteria, and Leite et al. (2009) [14] showed the antifungal activity of methanol and hexane extracts of avocado seeds, as well as larvicidal activity in vivo.

Pork consumption is estimated to increase by 105% from 2010 to 2050 due to the increase in the world population and in animal protein consumption [15]. The scientific community is currently aware of the environmental costs associated with pork production, which are mainly caused by the economic and environmental costs of producing and transporting feedstuffs, such as cereals and soybeans, which are primarily produced in North and South America [16,17]. The introduction of avocado seeds as an industrial by-product in the pork industry would facilitate progress in the circular economy and waste reduction. Despite scientific evidence on their beneficial effects, there are few studies evaluating the inclusion of avocado by-products in pig diets in terms of performance, nutrient digestibility, or carcass traits [9,18,19,20], and none specifically dealing with the inclusion of avocado seeds. Our hypothesis is that the inclusion of dried avocado seeds in the diet does not negatively affect performance and nutrient digestibility in growing pigs. The objective of the present study was to test the effects of dietary inclusion of dried avocado seeds on growing pig performance, nutrient digestibility, plasma metabolites, and carcass and meat traits. An energy value for avocado seed inclusion in pig diets will also be provided.

## 2. Materials and Methods

### 2.1. Animals, Diets, and Experimental Procedures

Twenty-four Landrace × Large White barrows with 23.8 ± 0.94 kg initial body weight (BW) and a similar age (78 ± 2 d), provided by Piensos Jiménez S. L. (Jaén, Spain), were randomly allocated to 3 experimental diets, and individually housed in 2 m^2^ pens in an environmentally controlled room (22 ± 1 °C). The experimental diets (8 pigs/treatment) were: control diet (CO; commercial diet based on barley, corn, and soybean meal) and two diets in which 100 or 200 g/kg of dried-milled avocado seeds from a local producer (Grupo La Caña, Motril, Granada, Spain) were added to partially replace (weight/weight) the CO diet (diets S10 and S20, respectively). The seeds were dried and milled before mixing with the rest of the dietary components. Diets were offered in pelleted form. The CO diet was a standard diet for growing pigs (Cereales MACOB S.L. Villanueva Mesía, Granada, Spain) covering all nutrient requirements [21]. Feed and water were freely available. The nutrient composition of experimental diets and dried-milled seeds appear in Table 1.

Pigs were weighed weekly, and individual intake was monitored daily by collecting the refused feed. Total tract apparent digestibility (TTAD) and nitrogen balance measurements were carried out after two weeks of adaptation to the experimental treatments and environmental conditions (33.8 ± 0.75 kg BW). Pigs were moved to individual metabolic cages in an environmentally controlled room (21 ± 1 °C) 2 days before starting the excreta collection. Whole feces and urine were daily collected for 3 days, weighed, and representative aliquots were stored at −20 °C until homogenization and analysis. Urine samples were collected on 50 mL 4.5M H_2_SO_4_. At the end of the experiments, samples of feces and urine were pooled per pig. Apparent energy digestibility, metabolizability, and nitrogen retention were determined as in the methods described in Palma-Granados et al. (2021) [22].

The experiment ended when pigs achieved approximately 40 kg BW, and the animals were slaughtered by exsanguination after electronarcosis. Blood samples were taken in EDTA-containing tubes, placed in an ice bath, and centrifuged at 1400× *g* at 4 °C for 20 min. Plasma was stored at −80 °C until analyses.

Measurements of midline backfat (first rib, last rib, minimum fat over the gluteus medius muscle) and leanness (gluteal thickness at the cranial end of the gluteus medius muscle) were performed in the left-half carcass. The weights and yields of primal cuts were determined 24 h postmortem, as described previously [23]. Muscle traits were determined in the right-half carcass, as described in Seiquer et al. (2019) [24]. Briefly, at 30 min post-mortem (p.m.), the pH values of longissimus (last rib level) were measured using a pH meter (HI 99163, Hanna Instruments, Cluj-Napoca, Romania) equipped with a penetration electrode (pH_30min_). Carcasses were placed at 4 °C for 24 h, and thereafter, pH was measured again (pH_24h_). Then, the longissimus muscle was dissected and separated from the carcasses. A 3 cm thick steak was then trimmed of visible fat and allowed to bloom for 15 min at 4 °C for color measurements, which were performed instrumentally using a Minolta CR-400 colorimeter (Konica Minolta Corp., Tokyo, Japan), in accordance with the CIE L*, a*, b* color system. Thereafter, samples were vacuum-packed and stored at −20 °C for chemical composition analysis. In addition, 1.5–2 cm steaks were cut from the longissimus muscle, trimmed of external fat and connective tissue, and used for drip loss determinations, as described in Seiquer et al. (2019) [24].

### 2.2. Sample Analyses

The chemical determinations of feeds, feces, and urine were as those described in Palma-Granados et al. (2021) [22]. Briefly, fecal samples were freeze-dried and ground before analysis. The dry matter and ash content were determined according to the Association of Official Analytical Chemists [25] in feeds and feces. The chemical determinations in muscle samples were as described in Seiquer et al. (2019) [24]. Nitrogen contents in feed, feces, muscle, and urine were analyzed using a LECO Truspec CN determinator (Leco Corporation, St. Joseph, MI, USA). Crude protein content was calculated using the factor of 6.25. Gross energy was analyzed in an isoperibolic bomb calorimeter (PARR 1356, Biometa, IL, USA). Urine samples were freeze-dried in a polyethylene sheet of known energy value, and their gross energy was obtained by difference. The lipid content of feeds was determined by ether extraction [25]. Amino acids in feeds were analyzed by HPLC (Alliance 2695 separation module; Waters Cromatrografía SA, Madrid, Spain) after protein hydrolysis with 6 N HCl at 110 °C for 24 h following the Waters Pico Tag method [26], as described in Palma-Granados et al. (2021) [22]. The acid detergent fiber in feeds was determined in an ANKOM220 Fiber Analyzer Unit (ANKOM Technology Corporation, Macedon, NY, USA), and were expressed inclusive of residual ash and lignin, determined by solubilization of cellulose with sulfuric acid, following Goering and van Soest (1970) [27]. The total extractable polyphenols in feeds was determined using the methodology described by Julkunen-Tiito (1985) [28]. Plasma metabolites (glucose, triglycerides, cholesterol, albumin, total proteins, ammonium, creatinine, lactate, urea, and uric acid), lactate dehydrogenase, alkaline phosphatase, alanine aminotransferase, aspartate aminotransferase, and γ-glutamyl enzymatic activities were determined in duplicate using a COBAS INTEGRA 400 analyzer (Roche Diagnostics GmbH, Mannheim, Germany).

### 2.3. Statistics

The statistical treatment of data was assessed by analysis of variance to check the effects of the addition of dried-milled avocado seeds to the basal diet on the parameters under study. An individual pig was the experimental unit. Mean differences were assessed by Tukey’s multiple-range test. The level of significance was set to 0.05 and a tendency of significance was considered for *p*-values between 0.05 and 0.10. Linear regressions using inclusion levels of dried-milled avocado seeds on the basal diet (0, 100, or 200 g/kg) as the independent variable and digestible or metabolizable energy content (kJ/g DM) as the dependent variable were carried out to calculate the energy value of dried avocado seeds for pigs. The statistical calculations were carried out using STATGRAPHICS Centurion XVI, version 16.1.18 (StatPoint Technologies Inc., Warrenton, VA, USA).

## 3. Results

In the following sections, animal performance, nutrient digestibility and nitrogen balance, carcass traits and meat quality determinations, and plasma biochemical parameters are shown.

### 3.1. Animal Performance

The main results regarding pig growth appear in Table 2. Pigs fed with a S20 diet had a lower BW at slaughter and average daily gain (ADG) than CO and S10 pigs (9.8 and 25% lower, respectively; *p* < 0.05). There were no differences in daily intake (*p* > 0.05). Feed efficiency, expressed either as gain/feed or gain/metabolizable energy intake (MEI), was depressed in S20 pigs (20 and 15%, respectively; *p* < 0.05).

### 3.2. Total Tract Apparent Nutrient Digestibility (TTAD), Nitrogen Balance, and Estimation of Energy Values for Dried Avocado Seeds

Results regarding the TTAD of nutrients and nitrogen balance are described in Table 3. Dry matter TTAD tended to decrease in S20 pigs (*p* = 0.071), and TTAD for organic matter and gross energy decreased in S20 pigs by 3 and 4%, respectively, compared to CO and S10 pigs (*p* < 0.05). Energy metabolizability was also lower in S20 pigs (*p* < 0.01), resulting in a decreased metabolizable energy intake in the S20 group (*p* < 0.01). Nitrogen TTAD and nitrogen retention were decreased in S20 pigs compared to the rest of the pigs (by 12 and 31%, respectively; *p* < 0.01). At the same time, the efficiency of retention of ingested nitrogen decreased by 16% in S20 pigs (*p* < 0.01), although no differences were detected for the efficiency of retention of digested nitrogen (*p* > 0.05).

To calculate the energy value of dried avocado seeds for pig diets, linear regressions using inclusion levels of dried avocado seeds on the basal diet (0, 100, or 200 g/kg) as the independent variable, and the digestible or metabolizable energy content of the whole diet (kJ/g DM) as the dependent variable, were performed. The following highly significant equations (*p* < 0.001) were obtained:

DE = 15.989 (±0.157) − 0.00452 (±0.00121) × S; n = 24; r2 = 64.9; s.e. = 0.45

ME = 15.421 (±0.154) − 0.00452 (±0.00117) × S; n = 24; r2 = 67.4; s.e. = 0.42

where DE, ME, and S are digestible energy (kJ/g DM), metabolizable energy (kJ/g DM), and inclusion level of dried-milled avocado seeds in the diet (g/kg), respectively. The intercept of these equations estimates the DE and ME content (kJ/g DM) of the basal diet (CO), the slope, and the decrease in energy value per g of increase in dried-milled avocado seeds in replacement of the CO diet (g/kg). When assuming S = 1000, the DE and ME contents of the dried avocado seeds used in the present trial are estimated as 11.5 and 10.9 kJ/g DM, respectively.

### 3.3. Body Components and Carcass Traits

The relative weight of body (g/100 g empty body weight) and carcass (g/100 g carcass) components of pigs are presented in Table 4. Many body components remained unaffected by the dietary addition of dried-milled avocado seeds. However, the relative weights of liver and small intestine increased in S20 pigs by 23% (*p* < 0.01) and 11% (*p* = 0.09). On the other hand, the relative weight of kidneys reduced in S10 compared to S20 pigs (14%; *p* < 0.05), spleen decreased in S20 compared to CO pigs (12%; *p* < 0.05), and stomach reduced in S20 vs. S10 pigs (13%; *p* < 0.05). Fat carcass components remained unchanged; meanwhile, some lean components decreased (sirloin by 13%; *p* < 0.01), or tended to decrease in S20 pigs (loin and muscle thickness, *p* = 0.09 for both).

### 3.4. Physical Quality Traits and Chemical Composition of Longissiumus Lumborum Muscle

**Figure 1:**
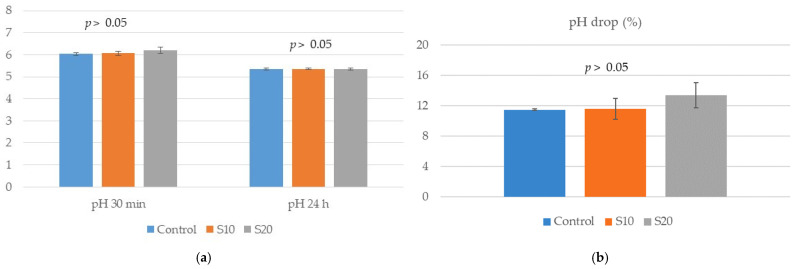
The pH at 30 min and 24 h post-mortem (**a**) and the corresponding pH fall (**b**) in longissimus lumborum muscle of growing pigs fed either a basal (control) diet or the control diet with 100 (S10) or 200 (S20) g of dried-milled avocado seed/kg added, respectively (eight pigs/treatment).

The muscle pH at 30 min and 24 h postmortem (Figure 1a) and pH drop between the two measurements (Figure 1b) showed no differences between experimental groups (*p* > 0.05). No differences were found in the chemical components and energy content of longissimus lumborum samples between groups (*p* > 0.05, Table 5). Similarly, muscle instrumental color parameters, brightness (L*), redness (a*), yellowness (b*), chroma index (C*) and hue angle (h°), showed no differences between the three groups (*p* > 0.05, Table 5).

**Figure 2:**
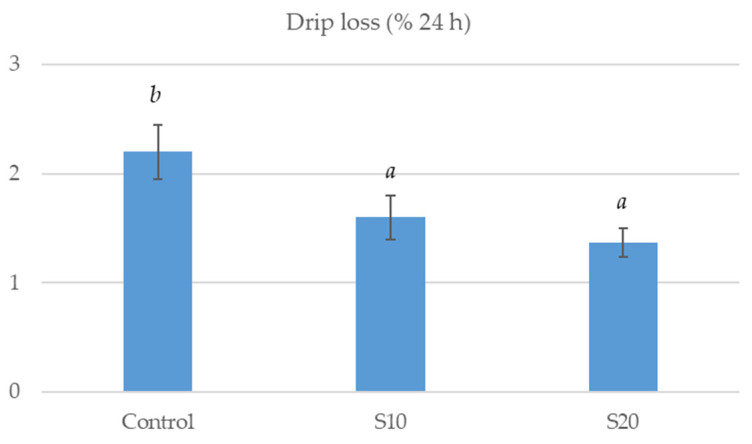
Water losses at 24 h post-mortem in longissimus lumborum muscle of growing pigs fed either a basal (control) diet or the control diet with 100 (S10) or 200 (S20) g of dried-milled avocado seed/kg added, respectively. Bars with different superscript differ (*p* < 0.05; eight pigs/treatment).

Nevertheless, muscle water losses 24 h post-mortem were lower in S10 and S20 compared to CO pigs (by 27–37%, *p* < 0.05; Figure 2) indicating a higher water retention capacity in pigs fed diets with dried-milled avocado seeds added.

### 3.5. Plasma Biochemical Parameters

Plasma metabolites showed no differences between treatments, apart from a greater creatinine concentration in S20 pigs (*p* < 0.01, Table 6), and a trend for higher total cholesterol and LDL cholesterol in S20 pigs compared to the other groups (0.05 ≤ *p* ≤ 0.10; Table 6). Enzyme activities were similar between the groups, except for a trend for lower alkaline phosphatase activity in pigs fed diets containing dried-milled avocado seeds (*p* = 0.079; Table 6).

## 4. Discussion

In EU countries, it has been estimated that the inclusion of food waste in pig diets could reduce the land used for pork production by 20% [29]. This issue is of particular interest for Mediterranean countries with large amounts of agroindustrial by-products, with a high potential for recycling in livestock diets and reducing food/feedstuff competition [30]. Spain has the largest pig herd in the EU [31], and is also the main European avocado producer. Therefore, there are opportunities for using avocado by-products generated during industrial processing in the national pork industry, contributing to waste reduction and the circular economy. We have evaluated the impact of inclusion of dried-milled avocado seeds in the diet on growth performance, nutrient digestibility, plasma metabolites, and carcass and meat traits in pigs, and provided an energy value for their inclusion in pig diets. Although scientific evidence highlights their potential benefits, there are few studies evaluating the incorporation of avocado by-products into pig diets regarding performance, nutrient digestibility, or carcass characteristics [9,18,19,20]. Moreover, no research has specifically focused on the inclusion of avocado seeds in diets for pigs.

In our study, the addition of dried-milled avocado seeds produced a slight reduction in crude protein and digestible energy (in S20 in particular) and an increase in acid detergent fiber, lignin, and total extractable polyphenol contents. The dried-milled seeds used in the present work showed similar crude protein, ash, and gross energy content, although a lower lipid content than in other reports (3.7 vs. 16%, ¸Talabi et al. 2016 [32]). Tugiyanti et al. (2019) [33] reported similar crude protein, total lipids and gross energy, and lower ash content than our study (2.7 vs. 5.2%). Other authors reported lower ash (1.34 vs. 5.2; [34]) or lipid content (1.1 vs. 3.7%; [35]). Differences in seed composition are probably related to local variations and seed processing.

Pig intake was not affected by the inclusion of up to 200 g dried-milled seed/kg diet, indicating good pig tolerance to seed addition in our study. Similarly, George et al. (2020) [11] reported no effect of dietary inclusion of avocado seeds on the feed intake of broilers, although inclusion levels were much lower than in the present case (0 to 1.5%). Fránquez et al. (2017) [18] reported decreased feed intake (by 30%) in pigs consuming diets containing 21% fresh whole avocado paste vs. control pigs (0% paste); however, diets were not isoenergetic, as the paste is rich in pulp with a high lipid and energy content, which probably satiated the pigs fed the paste-added diet earlier. In our study, despite similar feed intake, S20 pigs grew slower and showed reduced feed efficiency compared to the rest of pigs. The S10 group, in contrast, had similar growth and feed efficiency as CO pigs, with growth rates within the normal range for their BW [36]. Growing pigs fed isoenergetic diets including different proportions of avocado silage (0 to 5%) showed no differences in performance [37]; however, the physical–chemical characteristics of avocado silage (silage from paste after oil extraction) differed considerably from that of dried seed. In our work, apart from slower growth, the S20 group also presented reduced TTAD of nutrients, energy, and of nitrogen in particular, compared to the rest of pigs. The reduced nitrogen TTAD could be related to the higher polyphenol content of the S20 diet due to the higher seed proportion. The ingestion of polyphenolic compounds (such as tannins) can be beneficial due to their antimicrobial, antioxidant, and anti-inflammatory properties for the gut [38], although they can also negatively affect protein (amino acid) digestibility [39,40]. In this sense, phytochemical analyses of avocado seed extracts revealed the presence of saponins, oxalates, cyanogenic glycosides, and tannins, among others [41]. Grageola et al. (2019) [19] reported decreased fecal nutrient digestibility in growing pigs fed with diets supplemented with avocado paste (including pulp, seed, and peels), which was not observed with the addition of only avocado pulp [9], which might be related to the increased fiber and polyphenol contents in seeds and peels compared to the pulp.

On the other hand, the DE and ME values obtained for dried-milled avocado seeds in our trials (11.5 and 10.9 kJ/g DM, respectively) can be useful for the inclusion of this waste product in practical pig feeding, with both environmental and economic benefits, as mentioned above. However, it is desirable to extend the trials to other physiological phases such as gestating and lactating sows, in which even higher inclusion levels could be considered. As polyphenols in avocado seeds may negatively affect dietary nitrogen digestibility and nitrogen retention, further characterization of their biological effects is needed.

No major effects of dietary inclusion of dried-milled avocado seeds on the relative weights of body components were detected. However, lower proportions of lean carcass components in S20 pigs, together with increased creatinine plasma concentrations, could indicate higher muscle protein catabolism [42] in S20 pigs, despite unaltered urinary N excretion.

The chemical composition and physical and organoleptic properties of longissimus lumborum muscle were unchanged after dried avocado seed ingestion, which showed no detrimental effects on meat quality. Interestingly, a higher water retention capacity in pigs fed diets with dried avocado seeds added was detected, a trait that can be considered positive for the quality and organoleptic properties of the meat, as it is related to juiciness and tenderness [43]. Other authors found differences in longissimus chemical composition when 30% whole avocado paste was added to the diets of finishing pigs (higher protein and lower intramuscular fat, as well as differences in fatty acid composition [44]), possibly related to the higher lipid content of the whole avocado paste compared to that of the dried-milled seeds.

## 5. Conclusions

Under the conditions of our study, the voluntary feed intake of growing pigs was not affected by the addition up to 200 g dried-milled avocado seed/kg diet. Nevertheless, pig growth, nutrient and energy digestibility, and nitrogen retention were depressed with the highest level of inclusion of avocado seeds (200 g/kg diet). Polyphenols in seeds may have negative effects on nutrient digestibility, in particular on dietary nitrogen, and further characterization of their biological effects is needed. No deleterious effects on meat quality were detected; on the contrary, water holding capacity was enhanced with seed ingestion. An energy value of dried-milled avocado seed for inclusion in pig diets is provided (11.5 and 10.9 kJ/g DM, respectively), for which up to 100 g/kg could be safely added to practical diets for growing pigs, contributing to the value of this waste product.

## Figures and Tables

**Table 1:** Analyzed chemical composition of experimental diets and dried avocado seed, g/kg as fed ^1^.

	CO	S10	S20	Dried Seed
Dry matter	893	896	894	890
Crude protein	189	188	173	77.8
Lysine	11.2	10.8	11.0	3.7
Threonine	9.3	9.4	8.2	3.3
Methionine	4.1	3.8	3.9	1.2
Valine	9.9	9.6	9.4	4.6
Isoleucine	8.2	7.5	7.6	3.4
Total ashes	50.7	54.3	50.7	51.9
Lipids	47.6	44.9	46.2	37.1
Acid detergent fiber	51.2	64.0	60.0	96.1
Acid detergent lignin	5.3	16.3	18.6	56.8
Total extractable polyphenols	2.6	5.1	7.9	50
Gross energy (MJ/kg)	17.3	17.2	17.2	16.7
Digestible energy (MJ/kg) ^2^	14.1	14.0	13.5	10.2

^1^ CO: basal diet (g/kg: barley, 397; maize, 321; soybean meal, 226; lard, 25; NaCl, 5.0; CaHPO_4,_ 8.0; CaCO_3_, 6.3; vitamins and minerals, 4.0; L-Lys (98.5%), 3.9; L-Thr (98%), 2.0; DL-Met (99%), 1.4). S10 and S20: 100 or 200 g/kg of dried-milled avocado seeds added to partially replace (weight/weight) the CO diet, respectively. ^2^ Determined in present trials.

**Table 2:** Performance data of growing pigs fed either a basal (CO) diet or the CO diet added with 100 (S10) or 200 (S20) g of dried-milled avocado seeds/kg, respectively (eight pigs/treatment).

	Treatments				
	CO	S10	S20	SEM	*p*-Value ^1^
Initial BW, kg	23.7	23.8	23.8	0.94	0.993
Final BW, kg	41.2 ^b^	40.8 ^b^	37.0 ^a^	1.08	0.023
Intake, g DM/d	1599	1583	1481	60.0	0.324
ADG ^2^, g	500 ^b^	482 ^b^	368 ^a^	22.1	0.001
Gain/feed	0.313 ^b^	0.304 ^b^	0.248 ^a^	0.009	0.001
Gain/MEI ^3^, g/MJ	20.4 ^b^	20.2 ^b^	17.2 ^a^	0.618	0.002

^1^ Within a row, mean values showing different superscript differ (*p* < 0.05). ^2^ Average daily gain. ^3^ Metabolizable energy intake.

**Table 3:** Total tract apparent digestibility (TTAD) and nitrogen balance of growing pigs fed either a basal (CO) diet or the CO diet added with 100 (S10) or 200 (S20) g of dried-milled avocado seed/kg, respectively (eight pigs/treatment).

	Treatments				
	CO	S10	S20	SEM	*p*-Value ^1^
Mean BW, kg	37.9 ^b^	36.2 ^b^	32.7 ^a^	0.97	0.003
Dry matter TTAD	0.833	0.834	0.810	0.008	0.071
Organic matter TTAD	0.845 ^b^	0.845 ^b^	0.821 ^a^	0.008	0.047
Gross energy TTAD	0.816 ^b^	0.814 ^b^	0.784 ^a^	0.009	0.025
Energy metabolizability	0.791 ^b^	0.788 ^b^	0.752 ^a^	0.009	0.007
ME intake, MJ/day	20.4 ^b^	19.2 ^b^	16.8 ^a^	0.513	0.001
ME intake, kJ/kg^0.75^.day	1336 ^b^	1302 ^b^	1225 ^a^	23.6	0.008
ME, kJ/g DM	15.3 ^b^	15.1 ^b^	14.4 ^a^	0.17	0.003
N intake, g/day	45.0 ^b^	42.8 ^b^	36.1 ^a^	1.07	0.001
Nitrogen TTAD	0.819 ^b^	0.783 ^b^	0.701 ^a^	0.012	0.001
N retention, g/day	26.9 ^b^	24.4 ^b^	17.7 ^a^	0.95	0.001
N retention, g/kg^0.75^.day	1.76 ^b^	1.65 ^b^	1.30 ^a^	0.06	0.001
Retained N/ingested N	0.597 ^b^	0.569 ^b^	0.489 ^a^	0.017	0.001
Retained N/digested N	0.731	0.727	0.696	0.020	0.378

^1^ Within a row, mean values showing different superscript differ (*p* < 0.05).

**Table 4:** Body components (g/100 g empty body weight), carcass components (g/100 g carcass), and carcass linear measurements of growing pigs fed either a basal (CO) diet or a CO diet added with 100 (S10) or 200 (S20) g of dried-milled avocado seed/kg, respectively (eight pigs per treatment).

	Treatments				
	CO	S10	S20	SEM	*p*-Value ^1^
Body components					
Blood	4.91	4.99	5.29	0.128	0.445
Carcass	71.4	71.0	69.5	0.324	0.061
Total viscera	13.8	14.1	14.8	0.208	0.186
Heart	0.61	0.60	0.56	0.014	0.405
Liver	2.37 ^a^	2.50 ^a^	3.00 ^b^	0.061	0.001
Lungs	2.16	2.23	2.04	0.075	0.585
Kidneys	0.54 ^ab^	0.49 ^a^	0.57 ^b^	0.010	0.031
Spleen	0.26 ^b^	0.24 ^ab^	0.23 ^a^	0.005	0.028
Digestive tract	6.68	6.72	7.08	0.124	0.374
Stomach	1.05 ^ab^	1.08 ^b^	0.94 ^a^	0.021	0.027
Small intestine	3.36	3.37	3.75	0.078	0.091
Large intestine	2.27	2.27	2.4	0.045	0.418
Mesenteric fat	1.21	1.29	1.29	0.032	0.499
Carcass components					
Sirloin	1.51 ^b^	1.41 ^b^	1.27 ^a^	0.185	0.003
Loin	7.40	7.68	6.90	0.138	0.093
Butt lean	4.21 ^b^	3.46 ^a^	3.54 ^ab^	0.119	0.035
Backfat	2.21	1.81	1.71	0.098	0.113
Ribs	11.4	11.1	11.4	0.251	0.851
Ham	32.2	32.9	33.1	0.367	0.573
Shoulder	24.0	24.0	24.7	0.214	0.365
Belly	12.0	12.6	11.5	0.242	0.155
Spine	5.14	4.96	5.97	0.269	0.287
Backfat thickness, mm					
At gluteus medius muscle	6.5	7.6	6.4	0.364	0.343
At last rib	9.5	9.4	8.3	0.663	0.691
At first rib	15.9	16.9	16.0	0.701	0.819
Carcass length, cm	64.1	63.6	61.6	0.499	0.121
Muscle thickness ^2^, mm	45.4	44.1	40.1	0.957	0.088

^1^ Within a row, mean values showing different superscript differ (*p* < 0.05). ^2^ At gluteus medius muscle.

**Table 5:** Nutritional composition and color parameters of longissimus lumborum muscle of growing pigs fed either a basal (CO) diet or the CO diet with 100 (S10) or 200 (S20) g of dried-milled avocado seed/kg added, respectively (eight pigs/treatment).

	Treatments				
	CO	S10	S20	SEM	*p*-Value
Nutritional composition					
Protein, g/100 g	19.7	19.9	20.3	0.241	0.288
Intramuscular fat, g/100 g	3.10	2.62	2.76	0.194	0.226
Total ash, g/100 g	1.28	1.27	1.26	0.025	0.953
Energy kJ/100 g	544	534	547	10.4	0.666
Water, g/100 g	77.1	77.4	76.7	0.332	0.329
Color, CieLAB					
L*	38.9	38.9	37.2	0.348	0.272
a*	5.90	6.11	5.44	0.368	0.438
b*	4.23	4.56	4.11	0.358	0.680
C*	7.34	7.64	6.84	0.476	0.500
h°	36.2	36.8	36.1	1.54	0.940

**Table 6:** Plasma biochemical parameters of growing pigs fed either a basal (CO) diet or the CO diet with 100 (S10) or 200 (S20) g of dried-milled avocado seed/kg added, respectively (eight pigs/treatment).

	Treatments				
	CO	S10	S20	SEM	*p*-Value ^1^
Glucose, mg/100 mL	182	169	162	11.4	0.450
Triglycerides, mg/100 mL	42.6	34.3	42.1	4.00	0.285
Ammonia, µM/L	294	311	343	30.9	0.531
Lactate, mg/100 mL	159	168	171	23.7	0.937
Albumin, g/L	36.6	37.5	36.0	1.64	0.798
Total proteins, g/L	85.1	86.1	86.8	2.98	0.922
Total cholesterol, mg/100 mL	119	120	134	5.55	0.101
HDL cholesterol, mg/100 mL	50.9	47.9	49.5	2.09	0.599
LDL cholesterol, mg/100 mL	47.7	51.1	58.0	2.96	0.063
Creatinine, mg/100 mL	1.05 ^a^	1.11 ^a^	1.26 ^b^	0.04	0.002
Urea N, mg/100 mL	16.3	19.0	15.6	1.69	0.340
Uric acid, mg/100 mL	0.289	0.211	0.283	0.032	0.193
Alkaline phosphatase, U/L	196	122	147	22.1	0.079
Alanine transaminase, U/L	59.2	54.4	55.4	3.92	0.667
Aspartate aminotransferase, U/L	94.4	88.9	95.5	14.8	0.945
γ-Glutamyl transferase, U/L	150	111	94.9	20.1	0.159
Lactate dehydrogenase, U/L	2329	2188	2030	127	0.273

^1^ Within a row, mean values showing different superscript differ (*p* < 0.05).

## Data Availability

The datasets that support the findings of this study are available from the corresponding author upon request.
